# A phase I clinical and pharmacokinetic study of capecitabine (Xeloda®) and irinotecan combination therapy (XELIRI) in patients with metastatic gastrointestinal tumours

**DOI:** 10.1038/sj.bjc.6602354

**Published:** 2005-03-01

**Authors:** J P Delord, J Y Pierga, V Dieras, F Bertheault-Cvitkovic, F L Turpin, F Lokiec, I Lochon, E Chatelut, P Canal, R Guimbaud, D Mery-Mignard, X Cornen, Z Mouri, R Bugat

**Affiliations:** 1 Institut Claudius Regaud, 20-24, rue du Pont Saint Pierre, Toulouse 31052, France; 2 Institut Curie, Paris, France; 3 Centre René Huguenin, Saint-Cloud, France; 4 Aventis Pharmaceuticals, Paris, France; 5 Roche, Paris, France

**Keywords:** capecitabine, irinotecan, phase I, pharmacokinetic

## Abstract

Capecitabine is a highly active oral fluoropyrimidine that is an attractive alternative to 5-fluorouracil in colorectal cancer treatment. The current study, undertaken in 27 patients with gastrointestinal tumours, aimed to assess the toxicity and potential for significant pharmacokinetic interactions of a combination regimen incorporating capecitabine with 3-weekly irinotecan (XELIRI). Irinotecan (200 and 250 mg m^−2^) was administered as a 90-min infusion on day 1 in combination with escalating capecitabine doses (700–1250 mg m^−2^ twice daily) administered on days 2–15 of a 3-week treatment cycle. Pharmacokinetics were characterised on days 1 and 2 of the first two cycles. A total of 103 treatment cycles were administered. The principal dose-limiting toxicities were diarrhoea and neutropenia. Capecitabine 1150 mg m^−2^ twice daily with irinotecan 250 mg m^−2^ was identified as the maximum-tolerated dose and capecitabine 1000 mg m^−2^ with irinotecan 250 mg m^−2^ was identified as the recommended dose for further study. Analyses confirmed that there were no significant pharmacokinetic interactions between the two agents. The combination was clinically active, with complete and partial responses achieved in heavily pretreated patients. This study indicates that XELIRI is a potentially feasible and clinically active regimen in patients with advanced gastrointestinal cancer.

5-Fluorouracil (5-FU) has been the backbone of treatment for colorectal cancer (CRC) for more than 40 years. During this time, a number of schedules and regimens have been investigated. Irinotecan, a topoisomerase I inhibitor, is an effective treatment in patients with advanced/metastatic CRC unresponsive or resistant to 5-FU-based chemotherapy. Two pivotal, phase III studies in metastatic CRC, which demonstrated superior survival for irinotecan compared with best supportive care and infused 5-FU-based therapy, established irinotecan as a new agent for the second-line treatment of 5-FU-pretreated CRC (Cunningham *et al*, 1998; Rougier *et al*, 1998). In addition, combination of irinotecan with intravenous 5-FU/leucovorin (LV) was shown to significantly improve response rate, time to disease progression (TTP) and overall survival compared with 5-FU/LV alone in patients with previously untreated metastatic CRC (Douillard *et al*, 2000; Saltz *et al*, 2000). Recently, however, a high incidence of early treatment-related deaths was noted with the administration of the weekly irinotecan plus bolus intravenous 5-FU/LV schedule in this setting, and it has been suggested that continuous infusion 5-FU may be a safer option than bolus 5-FU for combination with irinotecan (Rothenberg *et al*, 2001).

Capecitabine (Xeloda; F Hoffmann-La Roche, Nutley, NJ, USA) is an oral fluoropyrimidine carbamate, which was rationally designed to mimic continuous infusion 5-FU. With capecitabine, 5-FU is generated preferentially in tumour tissue through high intratumoral concentrations of thymidine phosphorylase (TP) (Miwa *et al* 1998; Schüller *et al* 2000). Human pharmacokinetic studies, using validated methods for estimation of capecitabine and its metabolites, have shown that capecitabine is rapidly and almost completely absorbed through the gastrointestinal wall (Reigner *et al*, 2001). Capecitabine is metabolised to 5-FU via a three-step enzymatic cascade (Miwa *et al* 1998). In the first step, capecitabine is hydrolysed by hepatic carboxylesterase to 5′-deoxy-5-fluorocytidine (5′-DFCR), which is in turn converted by cytidine deaminase to 5′-deoxy-5-fluorouridine (5′-DFUR), the immediate precursor of 5-FU. The final step in the activation to 5-FU is mediated by TP, an enzyme that is highly active in tumour tissue compared with healthy tissue (Miwa *et al*, 1998).

Capecitabine is replacing 5-FU in CRC treatment. Two large, phase III trials including more than 1200 patients have demonstrated that, as first-line therapy for metastatic CRC, capecitabine achieves significantly superior response rates, with at least equivalent TTP and overall survival compared with 5-FU/LV (Hoff *et al*, 2001; Van Cutsem *et al*, 2001, 2004). Notably, the superior tumour response rate for capecitabine was particularly pronounced among the subpopulation of patients who had received prior adjuvant treatment with 5-FU. Capecitabine demonstrated an improved safety profile compared with 5-FU/LV and is associated with a very low incidence of alopecia and myelosuppression (Cassidy *et al*, 2002). The only adverse event occurring more frequently with capecitabine than with 5-FU/LV is hand–foot syndrome, which is easily managed by treatment interruption and dose reduction, and is never life threatening. Phase II studies of capecitabine in gastric and pancreatic cancer have also shown promising activity and a favourable safety profile (Cartwright *et al*, 2002; Hong *et al*, 2002; Koizumi *et al*, 2003).

In addition, capecitabine is a highly active component of combination treatment for metastatic CRC. A large international study has shown that capecitabine is highly active in combination with oxaliplatin, achieving a response rate of 55%, median TTP of 7.7 months and overall survival of 19.5 months as first-line therapy (Cassidy *et al*, 2004). The combination of capecitabine and irinotecan is supported by their different mechanisms of action. In preclinical evaluation, the combination of capecitabine and irinotecan demonstrated at least additive activity and was highly curative in tumour xenograft models (Cao *et al* 2001; Hapke *et al*, 2001). Additionally, capecitabine and irinotecan show only partial overlap of key toxicities. The predominant adverse events associated with irinotecan are neutropenia and diarrhoea. In a phase II study of 3-weekly irinotecan in patients with metastatic CRC, Grade 3/4 neutropenia and diarrhoea were observed in 40 and 26% of patients, respectively (Van Cutsem *et al*, 1999). Capecitabine is also associated with diarrhoea, but only minimal myelosuppression. In an integrated analysis of two large phase III trials of capecitabine in patients with metastatic CRC, Grade 3/4 diarrhoea was reported in 13% of patients, but Grade 3/4 neutropenia was observed in only 2% of patients (Cassidy *et al*, 2002). The significantly lower rates of diarrhoea and neutropenia occurring with capecitabine compared with 5-FU/LV (Mayo Clinic regimen) observed in these trials suggest that capecitabine may also be a better-tolerated combination partner for irinotecan.

The metabolic activation of both irinotecan and capecitabine is dependent on hepatic carboxylesterase activity. Irinotecan is cleaved by hepatic carboxylesterases to form the active metabolite SN-38, which is a potent inhibitor of topoisomerase 1 (Kuhn, 1998). As discussed above, the first step of the tumour-specific activation of capecitabine is conversion to the intermediate 5′-DFCR by hepatic carboxylesterase (Miwa *et al*, 1998). Consequently, there is potential for pharmacokinetic interaction between irinotecan and capecitabine.

There is therefore a clear rationale for investigating capecitabine in combination with irinotecan in patients with advanced gastrointestinal cancer. The current phase I clinical and pharmacokinetic study assessed the feasibility of combination therapy with capecitabine and irinotecan (XELIRI) in patients with advanced/metastatic gastrointestinal tumours. The primary objective of the study was to determine the maximum-tolerated dose (MTD) and dose-limiting toxicities (DLTs) of capecitabine, administered twice daily, on days 2–15 in combination with irinotecan, administered as a 90-min infusion, on day 1 of a 21-day treatment cycle. In addition, the study investigated whether significant pharmacokinetic interactions occur between the component agents, and evaluated the safety profile and antitumour activity of the XELIRI regimen.

## PATIENTS AND METHODS

This phase I, open-label, dose-escalation study of capecitabine and irinotecan combination therapy in patients with solid tumours of the gastrointestinal tract was conducted in three French Cancer Centres, in accordance with the International Good Practice principles and local ethical and regulatory requirements.

### Eligibility

The study included patients aged 18–75 years with histologically proven gastrointestinal tract cancer and no satisfactory options for further treatment. Patients were required to have a life expectancy ⩾3 months, Eastern Cooperative Oncology Group (ECOG) performance status 0–2, absolute neutrophil count ⩾2000 *μ*l^−1^, platelet count ⩾100 000 *μ*l^−1^, haemoglobin ⩾10 g dl^−1^, serum creatinine ⩽125 *μ*mol l^−1^, total bilirubin ⩽1.25 times the upper normal limit (UNL), transaminases ⩽3 times UNL, as well as prothrombin time and international normalised ratio within normal limits, and no evidence of severe infection, intestinal occlusion or subocclusion, or central nervous system metastasis. All patients provided written informed consent prior to study-specific screening procedures.

Patients were excluded from the study if they had received previous treatment with a topoisomerase inhibitor (irinotecan or other) or capecitabine, or had previously experienced allergic reactions to 5-FU. Additionally, patients were excluded if they had previously received total body irradiation or abdominopelvic radiation. Patients undergoing major abdominal surgery within 4 weeks of study entry or those with a history of serious cardiovascular disorder or renal, hepatic or metabolic disease that could potentially compromise the metabolism of the study drug were also excluded. Additional exclusion criteria included treatment with 5-FU within 4 weeks of study entry or with mitomycin C, nitrosourea compounds or extended radiation therapy within 6 weeks of study entry.

### Drug administration and dose escalation

Escalating doses of irinotecan (200–350 mg m^−2^) were administered in an intravenous infusion over 90 min on day 1 of a 3-weekly treatment cycle. Oral capecitabine (700–1250 mg m^−2^) was administered twice daily (approximately 12 h apart), within 30 min after a meal, on days 2–15. Antiemetic and antidiarrhoeal treatments and preventative therapy for irinotecan-induced early-onset anticholinergic syndrome were administered according to the policies at each centre.

At least three patients were recruited at each dose level and the dose was escalated when three patients had completed two treatment cycles without DLTs. If one or more of the three patients developed a DLT, the dose level was expanded to a total of at least six patients. If fewer than three of the six patients experienced a DLT, dose escalation was permitted, but if three or more of six patients experienced a DLT at a single dose level, that dose level was identified as the MTD. The dose level preceding the MTD was identified as the recommended dose and three additional patients were treated at this dose. No intrapatient dose escalation was permitted.

The maximum duration of treatment was six cycles. After this time, further treatment could be administered at the discretion of the investigator.

### Dose-limiting toxicities

Adverse events were classified according to the National Cancer Institute (NCI) Common Toxicity Criteria (CTC) Version 2.0. (1999). Any of the following toxicities occurring during the first two cycles of chemotherapy were considered dose limiting: any Grade 3 or 4 nonhaematologic toxicity (excluding alopecia and nausea); Grade 4 neutropenia or Grade 4 thrombocytopenia lasting for more than 7 days or accompanied by concomitant infection or bleeding, respectively; febrile neutropenia; nausea or vomiting preventing intake of capecitabine for at least 3 consecutive days; and any treatment-related adverse event causing a delay in the administration of the second treatment cycle.

### Patient and tumour evaluation

Patients were evaluated at baseline, on a weekly basis during the first two treatment cycles and at 3-weekly intervals thereafter. Evaluations included a complete clinical examination and recording of all adverse events, including severity and outcome. Complete blood counts (CBC) were performed at least twice weekly and blood chemistry analysis was performed weekly. A clinical tumour evaluation was performed during these visits, with the objective of detecting disease progression. A final evaluation, including a complete clinical examination, assessment of adverse events, CBC and blood chemistry analysis was conducted at the end of treatment.

In patients with measurable disease, tumour evaluation, based on World Health Organization (WHO) criteria, was performed at baseline, every 3 weeks for 6 weeks and at 9-weekly intervals thereafter. The best overall response was defined as the best response recorded from the start of treatment to disease progression. Complete responses (CR) and partial responses (PR) were confirmed by a second tumour assessment after 4 weeks. TTP was defined as the time from the start of treatment until disease progression.

### Pharmacokinetic evaluation

Pharmacokinetic evaluation was conducted during cycles 1 and 2. To determine the pharmacokinetics of irinotecan, blood was sampled for analysis of irinotecan and its metabolites 7-ethyl-10-hydroxycamptothecin (SN-38), SN-38 glucuronide and 7-ethyl-10-[4-*N*-(5-aminopeptanoic acid)-1-piperidino]-carbonyloxycamptothecin (APC) on day 1. Sampling times for irinotecan analysis included pretreatment and 3.0, 3.5, 9.5, 11.5, and 24.0 h after the start of the 90-min intravenous infusion. Plasma was recovered immediately after blood collection and the concentrations of irinotecan and its metabolites were measured by high-performance liquid chromatography (HPLC) as described previously (Rivory and Robert, 1995). Estimates of pharmacokinetic parameters of irinotecan and SN-38 were obtained by Bayesian analysis and POSTHOC option using the NONMEM program (version V, level 1.1, GloboMax Inc., Hanover, MD, USA) and a database of 67 previously evaluated samples (Chabot *et al*, 1995). The plasma area under the concentration–time curves (AUCs) of SN-38 glucoronide were determined using a limited-sampling method with stepwise linear regression, as recommended by Mick *et al* (1996). Plasma AUC of APC was determined by trapezoidal rule up to 24 h after the beginning of the irinotecan infusion (without extrapolation to infinity). The AUC values of irinotecan and its metabolites were compared between cycle 1 (before capecitabine administration) and cycle 2 (after a 2-week period of capecitabine treatment and 1-week wash-out) by using a paired Student's *t*-test.

For analysis of capecitabine and its metabolites, blood samples were collected on day 2 before capecitabine administration and at 0.5, 1.0, 2.0, 3.0, 4.0, 6.0, 8.0 and 12.0 h after administration. Plasma was recovered immediately after blood collection and concentrations of capecitabine and its metabolites 5′-DFCR, 5′-DFUR and 5-FU were measured using a validated reversed-phase HPLC technique with ultraviolet detection, slightly modified from the one described previously (Reigner *et al*, 1998). The AUC values were calculated using the linear trapezoidal rule. For capecitabine and its metabolites, the pharmacokinetic analysis was performed using the MicroPharm software (S Urien, Inserm-CRH, Saint-Cloud, France) and the Statview program (Abacus Concept Inc., USA) was used for the statistical analysis of the pharmacokinetic parameters.

## RESULTS

### Patient characteristics and disposition

A total of 27 patients were recruited to the study, from November 1999 to December 2001, of whom all were evaluable for safety, with 23 evaluable for tumour response. Patient characteristics are summarised in Table 1. The median age was 58 years (range 33–72 years) and the majority of patients (93%) had ECOG performance status 0 or 1. Most patients (78%) had CRC, four patients (15%) had gastric cancer and two patients (7%) had pancreatic cancer. With the exception of one tumour with epidermal histology, all tumours were adenocarcinomas and all patients had stage IV disease at study entry. Most patients had undergone prior surgery (85%) and had received previous chemotherapy, including 5-FU (81%).

### DLTs and recommended dose level

No DLTs occurred in patients treated at dose levels 1–3 (Table 2). At dose level 4 (capecitabine 1000 mg m^−2^ twice daily and irinotecan 250 mg m^−2^), one patient developed Grade 3 diarrhoea and Grade 4 neutropenia with septicaemia on day 6 of the first cycle. No further DLTs were experienced by the six patients treated at this dose level. Three of six patients treated at dose level 5 (capecitabine 1150 mg m^−2^ twice daily and irinotecan 250 mg m^−2^) developed DLTs: one patient experienced Grade 3 diarrhoea and abdominal pain by day 11 of the first cycle; one patient experienced Grade 3 diarrhoea and Grade 4 neutropenia on day 8 of the second cycle; and a further patient developed Grade 4 diarrhoea and Grade 3 vomiting by day 15 of the second cycle. To confirm the recommended dose, three additional patients were treated at dose level 4. As one patient was not evaluable for safety, a total of 10 patients were treated at this dose level. No further DLTs were observed. Therefore, the MTD was dose level 5 (capecitabine 1150 mg m^−2^ twice daily and irinotecan 250 mg m^−2^) and dose level 4 (capecitabine 1000 mg m^−2^ twice daily and irinotecan 250 mg m^−2^) was identified as the recommended dose for further phase II study. Overall, among the 10 patients treated at dose level 4, only one patient experienced a DLT.

### Safety profile

A total of 103 treatment cycles were administered to 27 patients and 16 patients received at least four cycles. The median duration of treatment was 2.8 months (range 0.07–16.1 months). Of the 10 patients treated at dose level 4, six patients received four or more treatment cycles. No cumulative toxicities were observed in patients completing more than four cycles.

The most frequent treatment-related adverse events were gastrointestinal disturbances, and the majority of cases were mild or moderate in intensity. Table 3 shows the incidence of Grade 3/4 adverse events by dose level. The only Grade 4 adverse events were diarrhoea in two patients (treated at dose level 5), nausea and vomiting, each in one patient (treated at dose level 4), and neutropenia in two patients (one treated at dose level 4, the other at dose level 5). Notably, only one patient (treated at dose level 3) experienced Grade 3 hand–foot syndrome.

### Pharmacokinetics

During cycle 1, plasma samples for pharmacokinetic studies were obtained from 23 patients on days 1 and 2. Pharmacokinetic data were evaluated in 23 patients during cycle 2. The mean AUC values for irinotecan and its metabolites are shown in Table 4. There were no significant differences between cycles 1 and 2 in AUC values for both irinotecan and SN-38. The AUC values of SN-38 glucuronide and APC were significantly different (*P*<0.05) between cycles 2 and 1 (% change from cycles 1 to 2: +15.3 and −19.3%, respectively). The mean AUC values for capecitabine, 5′-DFCR, 5′-DFUR and 5-FU are shown in Table 5. No significant differences were observed between cycles 1 and 2 at dose levels 1–5.

### Antitumour activity

Among 23 evaluable patients, an objective response to treatment was observed in two pretreated patients with CRC: a CR in one patient treated at dose level 4 and a PR in one patient treated at dose level 5 (Table 6). In addition, four patients achieved disease stabilisation. Among the eight evaluable patients treated at dose level 4 (the recommended dose for phase II evaluation), median TTP was 3.5 months (range 1.4–10.2 months).

## DISCUSSION

This study demonstrates that XELIRI (capecitabine plus irinotecan) is a feasible and promising new treatment for patients with metastatic gastrointestinal tumours. The recommended dosing schedule was identified as capecitabine 1000 mg m^−2^ twice daily on days 2–15 in combination with irinotecan 250 mg m^−2^, administered as a 90-min infusion, on day 1 of every 21-day cycle.

The benefits of combination therapy with irinotecan and 5-FU are well established, with phase III studies showing that the addition of irinotecan to intravenous 5-FU/LV significantly improves efficacy, including overall survival, compared with 5-FU/LV alone in patients with previously untreated metastatic CRC (Douillard *et al*, 2000; Saltz *et al*, 2000). It has been suggested that continuous infusion 5-FU may be a safer option in combination with irinotecan than bolus 5-FU (Rothenberg *et al*, 2001). Capecitabine is an oral agent providing chronic dosing that mimics continuous infusion 5-FU with a favourable safety profile compared with bolus intravenous 5-FU/LV (Hoff *et al*, 2001; Van Cutsem *et al*, 2001; Cassidy *et al*, 2002). Twice daily dosing with oral capecitabine offers numerous opportunities for dose adjustment during the treatment cycle, allowing safety to be readily optimised in patients receiving XELIRI. In addition, tumour-activated capecitabine may offer an enhanced therapeutic index via the generation of 5-FU preferentially in tumour. Replacement of infused 5-FU/LV with oral capecitabine is expected to simplify and improve the convenience of irinotecan/fluoropyrimidine combination therapy, because the XELIRI regimen requires only one clinic visit per 3-week cycle and avoids the inconvenience and potential complications associated with the protracted intravenous access required with infusional regimens.

The current phase I/pharmacokinetic study has demonstrated the feasibility of XELIRI. The MTD was identified as irinotecan 250 mg m^−2^ as a 90-min infusion on day 1 plus oral capecitabine 1150 mg m^−2^ twice daily on days 2–15, every 21 days. DLTS were assessed during the first two cycles of treatment in order to evaluate the potential for cumulative toxicity. The principal DLTs were diarrhoea and neutropenia, which are typical of fluoropyrimidine/irinotecan combinations (Saltz *et al*, 1996; Vanhoefer *et al*, 1999). The recommended dose is capecitabine 1000 mg m^−2^ twice daily on days 2–15 combined with irinotecan 250 mg m^−2^ administered as a 90-min infusion, on day 1 of every 21-day cycle. Among the 10 patients treated at this dose level, only one patient experienced a DLT.

Overall, the combination demonstrated a predictable safety profile, which was consistent with the known toxicity profiles of the single agents. The most commonly occurring adverse events were gastrointestinal disturbances, asthenia and neutropenia. However, Grade 1/2 diarrhoea occurred in the majority (75%) of patients experiencing this side effect, indicating that it was effectively managed in most patients by supportive measures and antidiarrhoeal medication. Similarly, Grade 1/2 neutropenia occurred in 75% of patients experiencing this side effect. Grade 3 and 4 neutropenia occurred in only four and two patients, respectively. Notably, Grade 3 hand–foot syndrome, a cutaneous side effect that is typical of infused fluoropyrimidines, was observed in only one patient.

Hepatic carboxylesterase is involved in the metabolism of capecitabine and irinotecan (Kuhn, 1998; Miwa *et al*, 1998). A pharmacokinetic evaluation was therefore performed to confirm the feasibility of administering capecitabine and irinotecan in combination and to determine the potential for interactions between these two agents. The similar AUC values for irinotecan and its metabolites during cycles 1 and 2 indicate that the administration of capecitabine does not impact significantly on either the AUC of irinotecan or SN-38 in this administration schedule. However, it should be noted that the drug-free period between treatment cycles precludes any definitive conclusions about the potential direct pharmacokinetic impact of capecitabine on irinotecan metabolism. Moreover, statistical differences were observed for the AUCs of nonactive metabolites (i.e. SN-38 glucuronide and APC), but the absolute change was less than 20%. Taken together, the changes in AUCs observed between cycles 1 and 2 (i.e. +16.4% for SN-38, +15.3% for SN-38 glucuronide and −19.3% for APC) indicate that the metabolism of irinotecan was modified between cycles without any change in the overall clearance of irinotecan. So far, no systemic changes in the pharmacokinetics of irinotecan and its metabolites have been described from cycles 1 to 2 when the agents are administered alone. Intraindividual variability of 14, 35 and 38% between cycles have been observed for plasma AUC of irinotecan, SN-38 and SN-38 glucuronide, respectively, but the number of cycles delivered did not significantly influence any of the pharmacokinetic parameters (Canal *et al*, 1996). Therefore, the changes in AUC observed in the current study are most likely attributable to the interaction of irinotecan with capecitabine. The trend we observed is consistent with the observation by Falcone *et al* (2001) that the AUC of SN-38 is increased when irinotecan infusion is preceded by 5-FU. In this study, the SN-38 AUC was 40% lower when irinotecan preceded 5-FU administration compared with the reverse sequence. However, whereas in the Falcone study 5-FU was administered immediately before irinotecan, in the current study, there was a 1-week wash-out period between administration of capecitabine and irinotecan. This schedule difference may explain the greater influence of 5-FU on irinotecan metabolism in the study by Falcone *et al* (2001), compared with the modest changes in the current study.

As expected, the AUC of capecitabine appears to increase linearly with dose escalation during both cycles 1 and 2. However, no significant differences in the AUC of capecitabine were observed between cycles 1 and 2; therefore, confirming that irinotecan does not have a major impact on the metabolism of capecitabine. This observation is consistent with the fact that no cumulative toxicity was observed at dose levels 4 and 5 in patients receiving more than two cycles.

Preliminary data from other pilot studies evaluating XELIRI (irinotecan 240–300 mg m^−2^ on day 1, or 100 mg m^−2^ on days 1 and 8, with intermittent oral capecitabine 1000 mg m^−2^ administered twice daily on days 1–14, every 3 weeks) in the first-line treatment of metastatic CRC have demonstrated promising activity with an acceptable safety profile (Bajetta *et al*, 2001; Kerr *et al*, 2002; Borner *et al*, 2003; Grothey *et al*, 2003). The current schedule affords convenience benefits compared with XELIRI regimens requiring weekly administration of irinotecan.

In a recent phase I study evaluating weekly intravenous irinotecan and capecitabine administered twice daily on days 1–14 of a 21-day cycle, the dose recommended for further evaluation was irinotecan 70 mg m^−2^ and capecitabine 1000 mg m^−2^ (Tewes *et al*, 2003). The study, which evaluated first-line XELIRI in patients with metastatic CRC, demonstrated good activity, with an overall response rate of 38%. It is worth noting that a UK/Dutch phase I study has identified a recommended regimen identical to that of the current study (Kerr *et al*, 2002). Preliminary data reported from a phase II trial show that this regimen (irinotecan 250 mg m^−2^ on day 1, followed by intermittent oral capecitabine 1000 mg m^−2^ twice daily for 14 days, every 3 weeks) is highly active as first-line therapy for metastatic CRC, achieving an objective response rate of 42% and median TTP of 7.1 months (Patt *et al*, 2003).

The response rate and median TTP achieved with XELIRI compare favourably with the results from randomised trials evaluating either infused or bolus 5-FU in combination with irinotecan as first-line therapy (Douillard *et al*, 2000; Saltz *et al*, 2000; Goldberg *et al*, 2003). In the current study, the vast majority of patients had received prior chemotherapy for treatment of advanced disease and more than one-third of patients had received multiple chemotherapy regimens. All chemotherapy-pretreated patients had received at least one 5-FU-based regimen. One patient treated at the recommended dose achieved a CR and a further patient treated at the MTD demonstrated a PR. Notably, both patients demonstrating a response to XELIRI had received prior treatment with 5-FU. Furthermore, in the patient demonstrating a PR, the current regimen was administered in the fourth-line setting. A further two patients experienced disease stabilisation at the recommended dose.

In conclusion, the XELIRI regimen is shown to be a feasible and clinically active chemotherapy regimen in patients with advanced gastrointestinal cancer. XELIRI offers a simplified regimen that is less cumbersome for patients and avoids the discomfort and complications associated with the central venous access required with continuous infusion 5-FU. The lack of pharmacokinetic interaction between capecitabine and irinotecan lends further support for evaluation of this XELIRI regimen in the phase II setting. Accordingly, the European Organization for the Research and Treatment of Cancer is currently evaluating XELIRI *vs* irinotecan plus infusional 5-FU/LV as first-line treatment for advanced CRC.

## Figures and Tables

**Table 1:** Baseline patient demographics (*n*=27)

**Parameter**	**No. (%)**
Median age, years (range)	58 (33–72)

*Gender*	
Male	18 (67)
Female	9 (33)
	
*ECOG performance status*	
0	13 (48)
1	12 (45)
2	2 (7)
	
*Primary tumour site*	
Colorectal	21 (78)
Gastric	4 (15)
Pancreas	2 (7)
	
*Histology*	
Adenocarcinoma	26 (96)
Squamous cell carcinoma	1 (4)
	
Median number of metastatic lesions (range)	3 (1–5)
*Prior treatment*	
Surgery	23 (85)
Radiotherapy	6 (22)
	
*No. of prior chemotherapy regimens* a	
0	5 (19)
1	13 (48)
2	3 (11)
>2	6 (22)

ECOG=Eastern Cooperative Oncology Group; 5-FU=5-fluorouracil.

a All regimens, including 5-FU.

**Table 2:** Incidence of DLTs during dose escalation

			**No. of patients**		
**Dose level**	**Capecitabine (mg m^−2^ twice daily)**	**Irinotecan (mg m^−2^)**	**Treated**	**With DLT**	**DLTs**
|	700	200	4	0	
2	850	200	3	0	
3	1000	200	4	0	
4	1000	250	10	|	Grade 3 diarrhoea, Grade 4 neutropenia/septicaemia
5	1150	250	6	3	Grade 3 diarrhoea/abdominal pain
					Grade 3 diarrhoea/Grade 4 neutropenia
					Grade 4 diarrhoea/Grade 3 vomiting

DLTs=dose-limiting toxicities.

**Table 3:** Grade 3/4 treatment-related adverse events

	**Adverse events: Grade 3/Grade 4**				
Dose level	1 (*n*=4)	2 (*n*=3)	3 (*n*=4)	4 (*n*=10)	5 (*n*=6)
Capecitabine (mg m^−2^ twice daily)	700	850	1000	1000	1150
Irinotecan (mg m^−2^)	200	200	200	250	250
					
Nausea	1/0	0/0	1/0	0/1	2/0
Diarrhoea	1/0	0/0	1/0	2/0	1/2
Vomiting	1/0	0/0	0/0	0/1	2/0
Stomatitis	0/0	0/0	0/0	0/0	1/0
Asthenia	1/0	0/0	1/0	0/0	0/0
Abdominal pain	0/0	0/0	0/0	0/0	1/0
Hand–foot syndrome	0/0	0/0	1/0	0/0	0/0
Leucopenia	1/0	0/0	0/0	1/0	1/0
Lymphopenia	0/0	0/0	0/0	0/0	2/0
Neutropenia	2/0	0/0	0/0	1/1	0/1

**Table 4:** Mean AUC values for irinotecan and its metabolites

	**AUC *μ*g ml^−1^ h (CV, %)**							
	**Irinotecan**		**SN-38**		**SN-38 glucuronide**		**APC**	
	**Cycle 1**	**Cycle 2**	**Cycle 1**	**Cycle 2**	**Cycle 1**	**Cycle 2**	**Cycle 1**	**Cycle 2**
Dose levels 1–3 (*n*=10)	11.6 (25)	11.9 (28)	0.518 (62)	0.539 (57)	0.838 (33)	0.909 (43)^a^	2.24 (59)^a^	2.25 (44)
Dose levels 4 and 5 (*n*=13)	14.9 (41)	16.0 (51)	0.587 (113)	0.640 (87)	0.876 (66)	1.043 (77)	1.61 (37)^b^	1.34 (40)^a^
Cycle 2 *vs* cycle 1 (% change±95% CI)	+6.2 (±10.1)		+16.4 (±20.2)		+15.3 (±13.0)		−19.3% (±15.3)	
*P*-value	NS		NS		<0.05		<0.05	

AUC=area under the curve; SN-38=7-ethyl-10-hydroxycamptothecin; APC=7-ethyl-10-[4-*N*-(5-aminopeptanoic acid)-1-piperidino]-carbonyloxycamptothecin; NS=not significant.

a Not available for one patient.

b Not available for two patients.

**Table 5:** Mean AUC values for capecitabine, 5-FU and its metabolites

	**AUC_(0–12 h)_ (s.d.) (mg ml^−1^ h)**							
	**Capecitabine**		**5-FU**		**5′-DFCR**		**5′-DFUR**	
**Capecitabine twice daily dose (mg m^−2^)**	**Cycle 1**	**Cycle 2**	**Cycle 1**	**Cycle 2**	**Cycle 1**	**Cycle 2**	**Cycle 1**	**Cycle 2**
700	3652 (1234)	3208 (820)	555 (157)	322 (130)	1929 N/A	2087 (910)	13 495 (4335)	11 179 (1300)
850	4343 (793)	4248 (1156)	407 (70)	465 (210)	1286 (586)	1890 (1164)	11 916 (1518)	10 887 (1914)
1000	7700 (3046)	7322 (3620)	478 (172)	598 (326)	6149 (3818)	7285 (3610)	14 341 (6769)	15 836 (3048)
1150	11 553 (5814)	11 188 (7377)	621 (156)	516 (189)	9967 (3526)	11 110 (2520)	18 001 (2572)	16 289 (5383)

AUC=area under the curve; s.d.=standard deviation; 5-FU=5-fluorouracil; DFCR=5′-deoxy-5-fluorocytidine; DFUR=5′-deoxy-5-fluorouridine; N/A=not available.

**Table 6:** Antitumour activity of XELIRI – best response

**Dose level**	**Irinotecan (mg m^−2^)**	**Capecitabine (mg m^−2^ twice daily)**	**CR**	**PR**	**Stable disease**	**Progressive disease**
1 (*n*=3)	200	700				3
2 (*n*=3)	200	850				3
3 (*n*=4)	200	1000			|	3
4 (*n*=8)	250	1000	|^a^		2	5
5 (*n*=5)	250	1150		|^b^	|	3
						
Total (*n*=23)			|	|	4	17

CR=complete response; PR=partial response.

Patient with colorectal cancer (CRC) who had previously demonstrated a partial response to 5-FU/oxaliplatin.

Patient with CRC who had previously demonstrated PR and CR, as well as disease stabilisation, after treatment with three previous 5-FU-based regimens.
